# The impact of COVID-19 pandemic on the indications of non-COVID-19 obstetric and gynecological admissions to the intensive care unit (ICU) and its overall consequences

**DOI:** 10.1038/s41598-023-46755-z

**Published:** 2023-11-20

**Authors:** Sevim Baltali, Aysun Firat

**Affiliations:** 1 Department of Anesthesiology and Reanimation, Istanbul Education and Research Hospital, University of Health Sciences Turkey, Istanbul, Turkey; 2 Department of Obstetrics and Gynecology, Istanbul Education and Research Hospital, University of Health Sciences Turkey, 34722 Istanbul, Turkey

**Keywords:** Diseases, Health care, Health occupations, Medical research

## Abstract

Since COVID-19 outbreak caused a substantial reduction in intensive care unit (ICU) bed capacity, a significant change in triaging ICU admissions has become necessary for obstetric and gynecologic (OG) patients, as well. In the present study, we aimed to analyze the patients admitted to ICU for non-COVID-19 OG pathologies to understand the probable effects of the pandemic on demographics, admission rates and indications, complications, and the overall outcome. Medical records of patients who were admitted to ICU for OG diseases between 2018 and 2022 were reviewed. This four-year time was divided into two equal periods; Group I (March 2018 to March 2020, before the pandemic starts) and Group II (March 2020 to March 2022, during pandemic). Demographics, indications for admissions to ICU, length of stay, acute physiology and chronic health evaluation II (APACHE-II) scores and the factors contributing to their morbidity and mortality were recorded. Chi-square Kolmogorov-Smirno and Shapiro–Wilk tests were used to compare the variables. p < 0.05 was considered statistically significant. 511 patients were in Group I (61.94%) and 314 in Group II (38.06%). Between 2020 and 2022, our ICU admitted 38.56% fewer OG inpatients, compared with the pre-pandemic period (p < 0.05). While number of patients with gynecological pathologies increased (50 vs 57%), obstetric patients’ admission to ICU decreased (49 vs 42%). In gynecologic patients, postoperative complications and sepsis showed a significant rise (57 vs 69% and 7 vs 12%, p < 0.05), and most were after oncological operations (81%). There was a significant rise in numbers of pregnancy-induced hypertension and placental pathologies (29 vs 36% and 41 vs 58%, p < 0.05). Outcome of obstetric patients in ICU was good (99% survival rate). Mortality was higher in gynecologic patients (4 vs 9%, p < 0.05), correlated with the increased APACHE-II score (8 vs 10, p < 0.05). Older age and oncologic operations were the primary factors increasing mortality. Length of stay in ICU prolonged in these patients, as well (1 vs 3 days, p < 0.05). Selection of priority patients by gynecologists and intensive care specialists in cooperation, and meticulous implementation of the rule of only accepting patients with strict indications may explain the change in OG admissions during the outbreak. These findings will question the accuracy of wider indications for ICU admissions in pre-pandemic period, and help in planning the policy for future post-pandemic days.

## Introduction

The coronavirus disease 2019 (COVID-19) pandemic is an ongoing world-wide health problem. The virus, severe acute respiratory syndrome coronavirus 2 (SARS-CoV-2), was first identified from an outbreak in China, in December 2019^1^. Attempts to contain the virus there failed, allowing it to spread to other areas of the world. Then, the World Health Organization (WHO) declared a pandemic on March 2020^2^. As of August 2022, the pandemic had caused more than 570 million infected cases and 6.4 million confirmed deaths, making it one of the deadliest pandemics in the history^3^. Increase in the number of cases and the fear of becoming infected with SARS-CoV-2 virus limited the social (e.g., contact with friends or family) and functional (e.g., exercising habits or daily activities) lives of all people, reverberating negatively on their health and well-being. Change in the perception of emergency situation due to limited resources available and uncertainty in the approach to non-COVID-19 critically ill patients have also emerged as new problems to be dealt with. Early evidence on how the first and second waves of the COVID-19 pandemic has affected non-COVID-19 obstetric and gynecological admissions (OGAs) to intensive care units (ICUs) is not known so far, since the COVID positive population with different demographics and multiple comorbidities participated more in the published studies^4–7^.

It has been well established that the most common diseases among OG patients requiring ICU admissions all around the world are postpartum or postoperative hemorrhage, hypertensive disorders of pregnancy, sepsis and malignancy^8,9^. It has also been known for long that the obstetric admissions to ICUs both in Europe and in Turkey were significantly reduced in the past two to three decades, as a result of the strict measures taken for the health of mother and baby^10,11^. Moreover, intensive care utilization by OG patients is relatively rare (5%-10%), compared to the general population^12^.

However, since the pandemic itself caused a substantial reduction in ICU bed capacity, the strict determination of the indications for ICU admission has become necessary for OG patients, as well. Therefore, to be familiar with the new demographic structure in OG patients and the differentiated ICU indications can help obstetricians and gynecologists and intensive care specialists to be ready for the new condition caused by COVID-19 outbreak.

In the present study, we aimed to analyze the patients admitted to ICU for non-COVID-19 OG pathologies to understand the probable effects of COVID-19 pandemic on demographics, admission rates and indications, complications, and the overall outcome.

## Materials and methods

Patients provided informed written consent to have data from their medical records used in research. The study was approved by our institutions’s Ethics’ Committee (SBU-IEAH-17.06.2022/207), and all methods were performed in accordance with the relevant guidelines and regulations**.** Medical records of patients who were admitted to ICU for OG diseases between 2018 and 2022 were reviewed. This four-year time was divided into two equal periods considering the start of COVID-19 pandemic from the beginning of March 2020. The groups were designed as; Group I (OGAs to ICU from March 2018 to March 2020, as control group before the pandemic starts) and Group II (OGAs to ICU from March 2020 to March 2022, as experimental group after the beginning of the pandemic).

Demographics, indications for admissions to ICU, length of stay, surgical procedures including operation notes and pathology reports, acute physiology and chronic health evaluation II (APACHE-II) scores and the factors contributing to their morbidity and mortality were recorded. All OG patients admitted to ICU had a negative real-time polymerase chain reaction (PCR) test for COVID-19. Patients with missing information in their files and those under the age of 18 were excluded.

APACHE-II score that used in our ICU is a severity-of-disease classification system, one of several ICU scoring systems^13^. It is applied within 24 h of admission of a patient; an integer score from 0 to 71 is computed based on several measurements: higher scores correspond to more severe disease and a higher risk of death. The point score is calculated from 12 admission physiologic variables comprising the acute physiology score (APS), the patient's age and chronic health status (Table 1). In the present study, APACHE-II score was used to describe the morbidity of a patient when comparing the outcome with other patients.

**Table 1:** APACHE-II scoring system.

A. Acute Physiology Score (measured within 24 h of admission)	
1. Oxygenation (mmHg)	
2. Body temperature (rectal, °C)	
3. Mean arterial pressure (MAP, mmHg)	
4. Blood pH	
5. Heart rate (HR, per minute)	
6. Respiratory rate (RR, per minute)	
7. Serum sodium level (Na, mEq/L)	
8. Serum potassium level (K, mEq/L)	
9. Creatinine (Cr, mg/dL, double point score for acute renal failure)	
10. Hematocrit (Hct, %)	
11. White blood cell (WBC) count (× 10^9^/L)	
12. Serum bicarbonate level (HCO_3_, mEq/L)	
Glasgow Coma Scale (GCS, 3 to 15 points)	
B. Age points	
Age (years)	Points
≤ 44	0
45–54	2
55–64	3
65–74	5
≥ 75	6
C. Chronic health points	
If the patient has a history of severe organ system insufficiency (liver cirrhosis, portal hypertension, class IV heart failure, severe respiratory disease, dialysis dependent, etc.) or is immunocompromised (chemotherapy, radiation, high dose steroid therapy, advanced leukemia, lymphoma, AIDS, etc.) assign points as follows:	
a. for nonoperative or emergency postoperative patients: 5 points	
b. for elective postoperative patients: 2 points	

A1–12; each 4 points, APACHE II score = Sum of A + B + C.

Complications and disease subgroup definitions were done using APACHE-II and traditional nomenculature, such as; bleeding (postpartum or postoperative inraabdominal or vaginal hemorrhage, requiring blood transfusion), cardiopulmonary diseases (serious cardiac or pulmonary events including angina, myocardial infarction, pulmonary emboli or arrest), placental pathology (placenta previa, abruptio placenta, etc.), and sepsis (a documented infection focus with systemic inflammatory response syndome/SIRS, with or without organ failure). Postoperative complications included all events after cesarean section or surgery, such as fluid and electrolyte imbalances, atelectasis, incisional seroma or hematoma without gross bleeding, surgical site infection (SSI) or intraabdominal abscess without sepsis, enterocutaneous fistula, etc.

Statistical package for social sciences (SPSS version 11.5) was used for the statistical analysis. Descriptive values were expressed as number (n), percentage (%), median or mean with standard deviation (SD). Chi-square test was used for nominal and categorical values, and Kolmogorov-Smirno and Shapiro–Wilk tests were used to compare the nonparametric variables. Group comparisons were done with Mann–Whitney U test to ascertain the group that cause the difference. P < 0.05 was considered statistically significant.

## Results

A total of 825 OG patients were admitted to ICU from 2018 to 2022; 511 were in Group I (61.94%) and 314 in Group II (38.06%). Between March 2020 and March 2022, our ICU admitted 38.56% fewer OG inpatients, compared with the pre-pandemic period (p < 0.05, Table 2). While the number of patients with gynecological pathologies increased (50.1% vs 57.64%, p < 0.05), obstetric patients’ admission to ICU decreased (49.9% vs 42.36%, p < 0.05) during the pandemic period.

**Table 2:** Change in the non-COVID-19 OGA to ICU before and after COVID-19 pandemic, and the overall otcome.

Parameters	Total (n = 825, 100%)		COVID pandemic			
			Before/Group I (n = 511, 61.94%)		After/Group II (n = 314, 38.06%)	
	n	%	n	%	n	%
Admission group						
Gynecologic (G)	437	52.96	256	50.1	181	57.64*
Obstetric (O)	388	47.03	255	49.9	133	42.36*
Indications for admission						
Bleeding	282	34.18	203	39.73	79	25.16*
G	80	28.37	50	19.53	30	16.57
O	202	71.63	153	60	49	36.84*
Hypertension	145	17.57	95	18.59	50	15.92
G	22	15.17	21	8.2	1	0.55*
O	123	91.03	74	29.02	49	36.84
Cardiopulmonary reasons	14	1.69	14	2.74	0	0.00*
G	13	92.86	13	5.08	0	0.00*
O	1	7.14	1	0.39	0	0.00
Placental pathology	62	7.51	26	5.09	36	11.46*
G	0	0	0	0	0	0
O	62	100	26	41.9	36	58.06*
Postoperative complications	275	33.33	150	29.35	125	39.81*
G	273	99.27	148	57.81	125	69.06*
O	2	0.73	2	0.78	0	0
Sepsis	47	5.69	23	4.5	24	7.64*
G	40	85.11	18	7.03	22	12.15*
O	7	14.89	5	1.96	2	1.5
Outcome						
Living	793	96.1	498	97.46	295	93.95*
G	407	51.32	244	95.31	163	90.06*
O	386	48.68	254	99.61	132	99.25
Exitus	32	3.8	13	2.54	19	6.05*
G	30	93.75	12	4.69	18	9.94*
O	2	6.25	1	0.39	1	0.75

*p < 0.05.

There was a significant change in the indications for OGAs to ICU, as well. In gynecological admissions, while the indications for hypertension and cardiopulmonary diseases decreased significantly (each, p < 0.05, Table 2), the patients with postoperative complications and sepsis showed a significant rise (57.81% vs 69.06% and 7.03% vs 12.15%, p < 0.05 for each; Table 2). Most of the postoperative complications were seen after oncological operations (n = 94/148, 63.5%, in pre-pandemic period vs n = 102/125, 81.6% during the pandemic, p < 0.05). However, in obstetric admissions, there was no statistical difference in cardiopulmonary and postoperative complications and in sepsis (each, p > 0.05). Moreover, indications for bleeding decreased significantly (p < 0.05). In obstetric patients, the most important finding in ICU admissions after pandemic is the significant rise in the numbers of pregnancy-induced hypertension and placental pathologies (29.02% vs 36.84% and 41.9% vs 58.06%, p < 0.05 for each; Table 2).

The outcome of obstetric patients in ICU was usually good (99% survival rate before and after the pandemic). However, mortality was seen to be higher in gynecological admissions during COVID period (4.69% vs 9.94%, p < 0.05). This finding was seen to be positively correlated with the increased APACHE-II score in gynecological admissions (mean, 8 vs 10 points, p < 0.05, Table 3), while it did not change significantly in obstetric patients (p > 0.05).

**Table 3:** The impact of pandemic on non-COVID OG patients admitted to ICU.

Parameters	COVID pandemic	
	Before (Group I)	After (Group II)
Age (years, range)	38 ± 16	42.5 ± 20*
Length of stay in ICU (days)	1 ± 0.8	3 ± 1.4*
Glasgow coma scale (points, mean)	14 ± 1.2	15 ± 0.2
APACHE-II (mean score, points)	7 ± 2	8 ± 2
Gynecologic	8 ± 2.5	10 ± 1.8*
Obstetric	6 ± 2	7 ± 3
Oxygenation (mmHg)	97 ± 10.1	99 ± 6.2
Body temperature (°C)	37.3 ± 2.1	37.6 ± 1.8
Mean arterial pressure (MAP, mmHg)	82 ± 26	85 ± 28
Blood pH	7.28 ± 0.34	7.31 ± 0.41
Heart rate (HR, per minute)	76 ± 28.1	81 ± 24
Respiratory rate (RR, per minute)	18 ± 6.4	18 ± 7.2
Serum Na level (mEq/L)	136 ± 11	135 ± 13
Serum K level (mEq/L)	4.2 ± 1.7	4.1 ± 2.6
Serum HCO_3_ level (mEq/L)	20.4 ± 6.3	18 ± 5.2
Serum Cr level (mg/dL)	0.56 ± 0.2	0.69 ± 0.2
Hematocrite (Htc, %)	33.2 ± 8.1	31.45 ± 6.4
White blood cell (WBC) count (× 10^9^/L)	13.73 ± 6.44	14.68 ± 7.91
Chronic health point (number, mean)**	1 ± 1	1.5 ± 1
Other biochemical values		
Serum lactate dehydrogenase (LDH, U/L)	258 ± 6	298 ± 9*
Serum alanine aminotransferase ALT, U/L)	13 ± 6	13.3 ± 7
Serum aspartate aminotransferase (AST, U/L)	21.93 ± 12	23.5 ± 14.4

*p < 0.05, **chronic health point (organ insufficiency; liver, pulmonary, renal, hearth, etc., immunocompromised state, obesity; body mass index/BMI score > 25).

Increased value in mean APACHE-II score in gynecological pathologies was mostly due to older patient admissions (mean 38 vs 42.5 years, p < 0.05, Table 3). There were no important changes in total acute physiology scores and chronic health points (p > 0.05 for each). Even anemia and leukocytosis were more prominent in Group II, there was no statistical significance (p > 0.05 for each). C-reactive protein (CRP), liver enzymes and renal function tests were also seen to be increased insignificantly (p > 0.05 for each). However, lactate dehydrogenase (LDH), a nonspecific serum marker, increased prominently (mean, 258 U/L vs 298 U/L, p < 0.05, Table 3), and the length of stay in ICU prolonged in the gynecological admissions (mean, 1 vs 3 days, p < 0.05).

## Discussion

The official worldwide deaths from COVID-19 pandemic surpassed 6 million, and it is still far from over^3^. The restrictions and global full or partial lockdowns have been initiated to slow down the spread of the virus and flatten the curve of the pandemic^14,15^. However, these measures had negative impacts in different strata of life, including the changes in accessibility and structure of health care delivery. From the time of early pandemic declared by WHO on March 2020, a moratorium on every kind of elective surgeries was placed to preserve the critical care resources. Since the high influx of patients with severe disease after the outbreak made the available medical staff and equipments insufficient to meet the needs of all patients, admission decisions to ICUs become more important. Therefore, the effects of COVID-19 outbreak on case volumes and ICU bed utilization have changed the traditional indications for admissions, disease control methods, demographics and overall outcomes in both COVID-19 and non-COVID-19 patients^16,17^. Health care utilization was affected by this major public health emergency, with elective high risk procedures and treatment for non-urgent conditions significantly cancelled or postponed^18,19^. The largest decrease was seen in preventive and primary care visits for common chronic conditions^18^. Furthermore, previous investigations have shown that even patients with life-threatening conditions may have avoided hospital admissions, possibly due to concerns regarding the exposure to SARS-CoV-2^20^. Substantial reductions in admissions and treatments for carcinoma, stroke and myocardial infarction were also reported^21,22^. A prolonged pandemic may also continue to exacerbate growing gender, social and economic inequalities with devastating consequences for those most at risk, since it has disproportionately impacted women, from reduced economic opportunities and decreased access to reproductive and maternal health care, especially in developing countries.

Although there are many post-pandemic publications on the change in demographics, treatment algorithms and outcomes of surgical patients admitted to both COVID and non-COVID wards and ICUs, our knowledge on OGAs only consists of clinical experience since OG patients constitute only 5 to 10% of general ICU population^12,16,17^. These studies reveal that there is over 90% reduction from baseline in the number of elective surgeries performed allowing 70 to 80% of surgical ICU beds to be available for COVID-19 positive patients^23,24^. In the present study, we analysed the patients admitted to ICU for non-COVID-19 OG pathologies as our hospital was not announced as a COVID-hospital in metropolitan Istanbul area, but we still had to be careful in reserving bed capacity in case of other COVID hospitals may overflow with patients. To our knowledge, this is the first study investigating the impact of COVID-19 outbreak on non-COVID-19 OGAs to ICUs, and according to our findings, our ICU admitted 38% fewer OG inpatients after March 2020.

Hemorrhage, hypertensive disorders of pregnancy, sepsis and malignancy are among the most common indications for the OGAs to ICUs^8,9^. However, preexisting medical conditions, cardiac diseases, respiratory disorders, and complicated operations are usually followed up in ICUs, as well. In a pre-pandemic study of Heinonen et al.^25^, published in 2002, the authors report that the most common diagnoses at ICU admission for the gynecological patients were postoperative haemorrhage (43%), infection (39%) and cardiovascular disease (30%). The mean duration of their stay in the ICU was 4.97 days and the mortality in the ICU was 0%. Other pre-pandemic studies of Sailaja et al.^26^ and Richa et al.^27^, published in 2019 and 2008, respectively, confirmed hypertensive disorders (24.2% vs 26%) and obstetric hemorrhage (23.1% vs 20%) as the most common obstetric admissions. In Richa et al.’ s ^27^ study, sepsis (26.7%) and preexisting medical problems (6.65%) were also ranked among the indications. According to our pre-pandemic admission indications; hemorrhage, hypertension and placental pathology in obstetric (60% vs 41% vs 29%, respectively), and postoperative complications and hemorrhage (57% vs 19%, respectively) in gynecological patients were detected as the leading causes. Sepsis and cardiopulmonary diseases (4.5% vs 2.7%, respectively) were also among the indications of OGAs to ICU, and the main disease groups were similar to the pre-pandemic literature, even the percentages differ probably due to regional population factors.

Since there is no similar study in our PubMed research of the English-written literature, we could compare the post-COVID-19 results with our own pre-outbreak data. The most important findings in the present study were the change in indications after outbreak as significant increases in admission ratios of pregnancy-induced hypertension and placental pathologies (36% and 58%, respectively) in obstetric, and postoperative complications and sepsis (69% and 12%, respectively) in gynecological patients. Significant decrease in admissions for hemorrhage in obstetric patients (from 60 to 36%) was also another interesting finding in the present study. In our opinion, priority given to oncological operations might explain the increase in postoperative complications seen in gynecologic patients. Since oncology patients were older, their APACHE-II scores were also higher. However, there were no important changes in their acute physiology scores and chronic health points. Similarly, we explain the decrease in hemorrhage in obstetric patients and in obstetric admissions to ICU partly by a result of the improvement in maternal and fetal care lately. Another contributing factor can be the effective use of postanesthesia care unit (PACU) and secondary care units located in the surgical wards, since most of the patients formerly admitted to ICUs were followed up in these units. Most importantly, the procedure of selecting priority patients by gynecologists and intensive care specialists in cooperation, and meticulous implementation of the rule of only accepting patients with strict indications may help to interpret the changes in OGAs during the outbreak. This has been achieved by changes in triage with denying ICU admissions to less-ill patients in order to accomodate those with postoperative complications and sepsis. This may also explain why bleeding patients are less well-represented since bleeding can typically be dealt with outside the ICU, but sepsis or ventilator dependency cannot.

The primary goal in ICU is to resuscitate patients and save their lives, and one of the most important secondary goals is to decrease the length of stay in order to improve the quality of medical care and reduce cost. However, prolongation of hospital stay in ICUs due to life-threatening diseases are increasing in the world^28^. According to literature, the mean length of stay in ICUs ranges from 1 to 28 days for most of the diseases including gynecological pathologies^29,30^. However, it is well known that elderly oncology patients with multiple comorbidities stay longer in ICUs^31,32^. In our study, comparatively shorter duration of stay in ICU was remarkable. It was on average only 3 days, even most of them underwent oncologic surgery. Because they were transferred to their OG wards as soon as their intensive care treatment ends.

The major limitation of our study is its retrospective design, which may cause difficulties in controlling for potential confounding bias. Moreover, we could not compare our single-center data with the literature since there was no similar articles in our web search. However, the present study investigating the impact of COVID-19 pandemic on the indications of non-COVID-19 OGAs to ICU will contribute to the current literature, since these findings remind the health professionals of the primary role of ICUs; admitting patients who really needs to be there. We also believe that our findings will question the accuracy of wider indications for OGAs to ICUs in pre-pandemic period, and help in planning the policy for future post-pandemic days.

## Data Availability

Data from this study are available upon request since there are legal restrictions (by Turkish Ministry of Health) on sharing data publicly. However, we anonymized the patients’ identities and protocol numbers on the system and saved whole data in Excel form. Data protection process and audit are supplied by Local Ethics’ Committe (ieahetikkurul@gmail.com, https://istanbuleah.saglik.gov.tr/TR-168206/kurul-uyeleri.html). The first author of the study will send them by email (s_baltali@yahoo.com).
